# Notes and News

**Published:** 1887-05-28

**Authors:** 


### Notes and News.

Collected and Communicated.

A new hospital is to be built for the parishes of Houston, Kilbarchan, Erskin, and Inchrinen, and the burgh of Johnstone, West Renfrew. The cost will be about £2,000.

The new Act of Parliament concerning the Aberdeen Charities now only awaits the Royal assent, and will come into operation on the 5th July.

At the last election of candidates for admission to the British Home for Incurables at Clapham there were eighty-five who were unsuccessful. A kind friend of this institution, who wishes to preserve an anonymity, sent a pound apiece fcr distribution to each of the unelected, in celebration of the year of Jubilee.

The twenty-first annual court of the Governors of the Victoria Hospital for Children was held on the 18th inst., under the presidency of the treasurer, Mr. Martin R. Smith, who, in the course of his address, remarked that the new out-patients' wing, which the Prince and Princess of Wales opened in June last, had been used during the past twelve months by nearly 38,000 little sufferers.

The committee of the Carmarthen Infirmary have issued their fortieth annual report, in which they refer with satisfaction to the continued efficiency and usefulness of the charity. During the past year sanitary improvements, involving an expenditure of nearly sixty pounds, were effected, and these will shortly be rendered complete by certain needed alterations in the ventilation. The infirmary has now invested capital to the amount of £10,761, the interest upon which, together with contributions from other sources, brought up the total income last year to £1,558, of which £530 was expended in purchase of Consols, so that the exchequer is in a most flourishing condition. The number of patients treated was 751, of whom 119 were in-patients.

On Friday last the annual meeting of the Council of the Metropolitan Hospital Sunday Fund was held at the Mansion House, Sir Sydney Waterlow presiding. The stimulus afforded last year by the Hospitals Week towards augmenting the collections in the churches of London is to be again adopted, but on a more extended scale, a Hospitals Fortnight having been arranged for this year. The collection will be made on Sunday, June 19, and during the whole of the preceding fortnight meetings will be held in all parts of London, enlisting the interest and sympathy of the public on behalf of our hospitals. It is expected that the speakers at these meetings will include some members of the Royal Family.

It is an old saying that one must go from home to hear the news. In the annual report of the Royal Infirmary, Aberdeen, just issued, "Mr. Saxon Snell, of London," is described as "the leading authority in the United Kingdom on the construction of hospitals We are aware that this gentlemen has built several workhouses and workhouse infirmaries, but we are not acquainted with a single modern hospital which has been built entirely from his plans, although he has made additions and extensions to existing buildings.

Have the trustees and managers of the Aberdeen Infirmary never heard of Professors de Chaumont and Marshall, of Captain Douglas Galton or Dr. Mouatt, who have some claim to be regarded as leading authorities, or of such hospital architects as Messrs. Waterhouse, Keith Young, Widdrington, Ingress Bell, and Gordon Smith? Each of these architects has shown to what extent he is entitled to be regarded as a leading authority, by designing hospitals which are worthy of study, and so affording evidence of a practical acquaintance with the points essential to the successful construction of a modern hospital. It is only fair that those who have really done the work should receive due public recognition as leading authorities, upon this and every other subject.

At a recent meeting of the General Committee of the Victoria Hospital at Bournemouth, it was reported that the total amount of the subscriptions to the fund for erecting a new hospital in celebration of the Queen's Jubilee was about £5,000, and that there was a like sum available from the old Dispensary Committee. The building will, it is estimated, cost about £7,000. The foundation stone of the new hospital will be laid on 21st June.

The names for the National Pension Fund are steadily growing in number, and lady superintendents and nurses have written from all parts of the country, from Edinburgh, from the Isle of Man, from Cornwall, from Norfolk, and from Wales; but despite this, the names sent in do not exceed two hundred to the present date, and ten times that number must be registered before steps can be taken to launch this Fund upon a sound financial basis. We shall be glad to send notices or cards, to be suspended in the nurses' rooms of any hospital or nursing institution, on receiving a request to that effect from the lady superintendent. The authorities of the Nightingale Fund are disposed to support the proposal for the establishment of a National Pension Fund, provided a sufficient number of nurses and matrons declare themselves to be favourable to the movement, and a scheme can be formulated which is calculated to meet to the fullest extent the requirements and needs of those who would be likely to join. Everyone, therefore, who desires to see the National Pension Fund speedily established, should use their influence with any of their acquaintances who are engaged in nursing or hospital work, with the object of inducing them to send in their names to Mr. Collins, the Secretary of The Hospitals Association, Norfolk House, London, W.C., without further delay.

THE following vacancies are announced this week, the amount of the salary being placed in each case after the name of the institution :—

**Honorary Medical Officers.**

Brighton Throat and Ear Dispensary. Dental Surgeon. Licentiate.

**Resident Medical Officers.**

Brighton Alexandra Hospital. Assistant Physician. Doubly-qualified.
Kilburn Dispensary. House Surgeon.—£100.
University College. Resident Medical Officer in the Hospital.

**Non-resident Medical Officers.**

Ongar Union. Medical Officer and Public Vaccinator.—£75.
Tawd Vale Accident Society, Skelmersdale.—£150.
Wilton Union, Salisbury. Medical Officer and Public Vaccinator.—£100.

**Matrons, etc.**

Eastern Fever Hospital.—£100.
Glasgow Infirmary. Night Superintendent.—£35.
Islington Fever Hospital. Night Superintendent.—£30.

**Trained Nurses.**

Bolton Infirmary. Charge Nurse, and two Assistants.
Chester Deaconesses' Institution. Two.—£24.
Eastern Fever Hospital. Several.—£30.
Leeds Hospital for Women and Children.—£20.
Manchester Hospital for Incurables. Head.—£30.
Middlesbrough Infirmary. Charge Nurse for Male Ward.—£25.
Worcester Nurses' Institution. Several.

**Probationers.**

Bolton Infirmary. One.
Westhulme Hospital, Oldham. One.—£16.

---

AN excellent performance was given on the 17th inst. at St. George's Hall, Langham Place, by the Irving Amateur Dramatic Club, in aid of the Chelsea Hospital for Women. The pieces selected for presentation were a tragic drama by R. H. Horne, entitled "The Death of Marlowe," and a three-act comedy by Sydney Grundy, "The Silver Shield." Miss Webster, by kind permission of Messrs. Hare and Kendal, played the leading *rôle* of Alma Blake in the latter piece, her acting eliciting marked expressions of popular approval. The whole of the characters were intelligently and ably played, and we sincerely hope that the performance will be found to have proved equally successful from a pecuniary, as it undoubtedly was from a histrionic point of view. During the evening Mr. Norfolk Megone's Bijou Orchestra gave a pleasing selection of airs. The hall was crowded by a fashionable company.

---

THE annual report of the Hospital of St. John and St. Elizabeth, Great Ormond Street, for 1886, bears witness to the patient and unostentatious work which this institution accomplishes. "The object of our hospital," writes the Mother Prioress, "is to receive cases of long-standing disease, which for that reason are not retained at usual hospitals, and which require lengthened treatment, if they are capable of being restored, and often linger for a protracted period, if their malady is necessarily fatal." Last year, of the eighty-two cases admitted, twenty-six died and fifty-six were discharged cured or improved. Though the institution is a Catholic one, the religious feelings of patients of other creeds are scrupulously observed. Among the legacies received last year was one of £1,000, from the late Mrs. Richardson ; Mrs. Blumenthal gave a donation of £500, for the perpetual endowment of a bed ; and the Hospital Sunday Fund contributed £156 5*s*.

THE Hospital for Sick Children in Great Ormond Street has been passing through a period of great pecuniary difficulty, and though the cloud has lifted, it has by no means passed away. In 1884 a heavy loan of £1,500 had to be obtained to meet special sanitary expenses, and the subscriptions, etc., falling off, this incubus of debt was swollen during the next twelve months to £2,500. Before taking the regretable step of closing one or more of the wards, the committee made an urgent appeal to the benevolent public. This call found a generous response, two anonymous donors sending £1,000 and £500 respectively. The hospital, which is the oldest institution in London devoted solely to the diseases and accidents of children, has just completed its thirty-fifth year. Last year the in-patients admitted numbered 1,094, and the out-patients 15,066. At the Cromwell House branch at Highgate there were 120 chronic cases and 143 convalescents received. The new wing to complete the building, towards which English children all over the globe are contributing their little mites, is felt to be a very pressing want.

---

SOME few months ago we drew the attention of our readers to the Cheyne Hospital for Sick and Incurable Children, a little institution—almost unique of its kind—housed in a couple of old and semi-dilapidated residences in Cheyne Walk. For ten years the Cheyne Hospital for Sick Children has carried on its noble work under conditions—as far as the premises go—by no means of the happiest description. The rebuilding of the Hospital, to which we alluded, has now been decided upon by the committee, upon the urgent recommendation of the medical staff. An excellent freehold site, facing the river, in Cheyne Walk, has been obtained, and the building fund is progressing rapidly. The Hospital will cost about £6,000, towards which the following donations (amongst many smaller ones) have been made :—A. B. F. M., £1,000 ; A Friend, £500 ; Wickham Flower, Esq., 200 guineas ; Court of Common Council, 100 guineas ; M., £100 ; Messrs. Williams, Deacon, and Co., £100 ; A. G. Crowder, Esq., £60 ; The Fishmongers', Mercers', and Grocers' Companies, 50 guineas each ; and the Goldsmiths' Company, £50. Mr. H. Kemp-Welsh, of 46, Cheyne Walk, Chelsea, is the honorary treasurer to the fund.

---

THE fiftieth annual report of the National Orthopædic Hospital, 234, Great Portland Street, Regent's Park, has just been issued. During the fifty years the institution has been at work more than sixty thousand patients, most of them children of tender years, have undergone treatment within its walls. The present buildings are inadequate to the demands made upon them, and the committee have started a special appeal for donations to a building fund. For this object the sums at present received or promised amount to £1,860. During last year 109 in-patients were discharged from the hospital either cured or greatly relieved, whilst in the out-patient department 1,002 new cases and renewals received attention. The receipts for the year amounted to £1,237, and the expenditure to £1,072. Of the former item, £720 was received from or on behalf of in-patients.

The following sums have been recently received by the various Medical Charities, the name of the donor or the source from which they are obtained being given in each instance after the name of the Institution :—

**Donations.**

British Home for Incurables, Clapham. Messrs. Baring Bros., £10 10*s.*
Convalescent Homes for Little Children (Lancing and Ramsgate). Mr. Hy. M. Harvey, £10.
Hospital for Epilepsy and Paralysis, etc., Regent's Park. The Grocers' Company, £50.
National Hospital for Consumption. Mr. W. D. James, £50.
National Truss Society. The Grocers' Company, £50.
North London Consumption Hospital. Mr. H. Sutton, £31 10*s.*
North London Hospital. Mr. C. Gooden, £10 10*s.* Mr. Geo. C. Raphall, £10. Mr. James Hochee, £10 10*s.* Mr. Hy. Lucas, £10 10*s.*
Royal Free Hospital. Churchwardens of the Parish of St. Dionis Backchurch, Fenchurch Street, £25.
The Hospitals Association. The Drapers' Company, £20.

**Entertainments.**

Home for Incurable Children, Maida Vale. Mrs. Walter Weblyn, £10, being the proceeds of an amateur entertainment given at her house.

---

THE report of the Gloucester General Infirmary and Gloucestershire Eye Institution for 1886 refers with satisfaction to the increased efficiency of the institution, caused by the completion of the structural improvements in the north wing last year. Financially the condition of affairs is hardly so satisfactory, as the accounts published show a deficit of nearly £1,200. The report states, however, that "the occasional funds have been large, and it is from this source that the deficiency in the balance of the year has been met," so we suppose the managers are satisfied. It would be more satisfactory, however, in a published report, if the reader were told what sources of income come under the head of "occasional funds," and what total these funds amounted to. During 1886, 1,229 in- and 5,648 out-patients received treatment, and 987 of the latter were eye-cases.

---

WHITECHAPEL, in the neighbourhood of the London Hospital, was quite *en fête* last Saturday. The occasion was the visit to that time-honoured institution of the Prince and Princess of Wales, who had promised to open the new Nursing Home and Medical College buildings. The Prince and Princess, accompanied by their two daughters, the Princesses Louise and Victoria, and by the Crown Prince of Denmark, arrived at the hospital shortly after five. The main entrance-hall was very prettily decorated with flowers. Here a procession was formed, and a move made to the Queen Victoria wards at the eastern end of the hospital. The little occupants of the cots, some sitting up, others too ill to make that effort, greatly interested the Princess and her daughters, and the children seemed fully to reciprocate the sympathetic smiles of the Royal ladies. The Princess of Wales visited all the cots, speaking or smiling to the little sufferers, distributing piecemeal the beautiful bouquets she carried. While the Princess was busied talking to the little children, the Prince visited an isolated part of the hospital occupied by a young man whose awful deformities have gained for him the nickname of the "elephant man." Mr. F. C. Carr Gomm, the Chairman of the hospital, recently made an effort to find a suitable home for this terribly afflicted man, but, though money for his support was readily forthcoming, no institution could be found to receive him. As a very special case, the authorities of the London Hospital have consented to keep him, and the poor man appears very grateful for the kindness shown him. Leaving the children's ward, the Royal party proceeded through the Gloucester ward (a male accident ward), the Princess again stopping to speak to some of the sufferers. Proceeding to the Nursing Home, the distinguished visitors were received by Miss Lückes, the matron, in the sisters' and nurses' dining-room. The Princess of Wales having declared the Nursing Home open, the Royal party was conducted to the Medical College. The guests met in the library, where the Duke of Cambridge, as president of the hospital, read an address to the Prince and Princess, the former delivering a gracious reply. Dr. Langdon Down, the senior physician, thanked the Prince and Princess for their presence. The Prince of Wales then declared the College open. After the choir had sung "Rule Britannia," the Royal party proceeded to the board-room, where refreshments were served.

---

ON the 19th inst. a grand "variety" entertainment took place at St. George's Hall in aid of the Hospital for Epilepsy, Regent's Park. The programme included two short comedies and an exhibition of Mrs. Jarley's popular "waxworks." An interesting feature of the evening was a gavotte, danced by four ladies and four gentlemen, in costumes of the period. There was a large attendance. The entertainment was the first of a series which the friends of this institution are arranging with a view to raising a sum of £2,500 for the purchase of the lease and the extinction of the debt.

---

THE London Homœopathic Hospital propose, as a memorial of the Jubilee, to build a Convalescent Home in connection with the institution. In furtherance of this object, the Duke of Westminster has kindly granted the use of the picture-gallery of Grosvenor House for the purposes of a concert, under the leadership of Mr. Wilhelm Ganz and Signor Adelmann. A number of distinguished *artistes* have promised their assistance. The concert will take place to-day (Saturday) at three o'clock.

---

THE City Provident Dispensary and Surgical Appliance Association is the only provident dispensary which the City of London possesses, and its practical value has been well tested during its past career of ten years. It has now migrated from its old quarters in Aldersgate Street to much more convenient premises at 4, Little Britain, the inauguration of which was celebrated last Monday. The object of the City Dispensary is to provide medical and surgical advice with medicine to the working classes at fees proportionate to their means. During the past ten years 27,237 patients have attended, and 6,753 surgical appliances and 7,552 instruments have been supplied. For the past two or three years the dispensary has been entirely self-supporting.

